# Post-Operative Cognitive Dysfunction in Elderly Patients Receiving Propofol Sedation for Gastrointestinal Endoscopies: An Observational Study Utilizing Processed Electroencephalography

**DOI:** 10.7759/cureus.46588

**Published:** 2023-10-06

**Authors:** Christopher P Potestio, John Dibato, Kelly Bolkus, Ahmed Awad, Umashanger Thayasivam, Avish Patel, Anshel Bright, Ludmil V Mitrev

**Affiliations:** 1 Department of Anesthesiology, Cooper Medical School of Rowan University, Camden, USA; 2 Department of Clinical Biostatistics, Cooper Medical School of Rowan University, Camden, USA; 3 Department of Anesthesiology, Cooper University Health Care, Camden, USA; 4 Department of Anesthesiology, Cooper University Hospital, Camden, USA; 5 Department of Mathematics, Rowan University, Camden, USA

**Keywords:** processed eeg, non-operating room anesthesia, procedural sedation, geriatric anesthesia, postoperative cognitive dysfunction

## Abstract

Background: Propofol sedation is commonly administered during gastrointestinal (GI) procedures. The Patient State Index (PSI) is a processed electroencephalography (EEG) parameter obtained with the SedLine^®^ Sedation Monitoring system (Masimo Corporation, Irvine, CA). When used to objectively assess the patient’s level of consciousness, PSI may provide a more effective, safer titration of sedation during GI procedures. We hypothesize that having more or longer episodes of deep sedation as assessed by PSI (i.e., PSI<26) would correlate with developing new-onset or worsening post-operative cognitive dysfunction (POCD).

Methods: This was a pragmatic, double-blinded observational study of 400 patients aged ≥65 years undergoing upper GI endoscopy, lower GI endoscopy, or a combined procedure utilizing propofol sedation at a tertiary-care [A1] academic medical center. The patients were monitored with the SedLine^®^ Brain Function Monitor, software version 2 (Masimo Corporation, Irvine, CA), throughout the case, starting at baseline (i.e., before administration of propofol) and stopping at case end. We assessed the subjects’ cognitive function via an in-person interview at baseline (pre-procedure) and telephone interviews at 1, 7 (±1), and 90 days after study enrollment. Cognitive function was assessed by administering the short blessed test (SBT), which is a validated brief cognitive screening appropriate for in-person and telephone administration.

Results: The correlations between the change in SBT score and the pre-defined parameters of PSI were not significant (all *p*-values >5%). There was a significant drop in SBT scores on day seven. Higher age was also significantly associated with a drop in SBT from baseline. Deep sedation, as evidenced by the number of times PSI was lower than 26, was not predictive of the change in SBT, nor was gender, total propofol dose, or vasoactive drug use during the procedure.

Conclusions: The observed incidence of POCD after GI procedures with propofol sedation was low (1.3% at seven days and 2.95% at 90 days) and lower than at the baseline. Age was associated with a greater average decline in SBT score, although the absolute change was small (−0.067 per year of age increase). Deeper sedation, as documented by the PSI score, was not associated with a change in POCD measured with the SBT.

## Introduction

Propofol sedation is commonly administered during gastrointestinal (GI) endoscopies. A patient’s level of consciousness can be monitored during sedation with processed electroencephalography (EEG). Implementation of processed EEG to objectively assess the patient’s level of consciousness provides a more effective, and possibly safer, titration of sedation during these procedures.

Liu et al. reported that between 2003 and 2009, 1,573,726 privately insured patients had either an upper gastrointestinal endoscopy or a colonoscopy as outpatients with the administration of anesthesia in the United States [1]. The corresponding number was 302,263 patients in the Medicare fee-for-service population. Most of the patients in the Medicare group were older than 65 years, whereas the ages of the privately insured patients ranged from 46 to 65 years. These results indicate that the market for propofol sedation and monitoring in the United States is significant.

Data regarding the incidence of postoperative cognitive dysfunction (POCD) in patients undergoing gastroenterological endoscopies are lacking. In a small open-label randomized control trial of patients undergoing outpatient colonoscopy, Padmanabhan et al. found that patients’ cognitive function was worse at discharge than at baseline, but cognitive impairment did not differ between those receiving propofol only and those receiving fentanyl and midazolam in addition to propofol infusion [2]. In a single-center cohort study, Chua et al. found that more than 50% of geriatric patients undergoing outpatient endoscopy procedures exhibited cognitive dysfunction upon discharge [3]. In another study of patients undergoing sedation for coronary angiography, a procedure with stress levels similar to those in GI endoscopy, and baseline comparator groups of patients who did not undergo any procedure but were otherwise matched in terms of controls, the results suggested that the incidence of cognitive dysfunction in the sedation group was 21% at 90 days, whereas it was 0% in the non-intervention control group [4].

The level of sedation and its effect on cognitive function have been studied in larger and more complicated procedures. In patients undergoing hip fracture repair, randomization into lighter sedation or deeper sedation groups had no impact on postoperative cognitive function [5]. Another study reported a 24% incidence of POCD in patients undergoing major noncardiac surgery, with the incidence increasing to 47% at one month, decreasing to 23% at two months, and further decreasing to 16% at six months [6]. The interval from the time of surgery and the degree of cognitive decline vary across studies [6-8]. Nevertheless, addressing postoperative cognitive function is imperative because POCD after noncardiac surgery in the elderly has been associated with increased mortality, the risk of leaving the labor market prematurely, and dependency on social transfer payments [9].

We designed a pragmatic, observational cohort study with the following aims: to document the incidence of POCD in patients undergoing GI endoscopies with propofol sedation and to determine whether the Patient State Index (PSI) processed EEG parameter obtained with the SedLine® Sedation Monitoring system (Masimo Corporation, Irvine, CA) is associated with, and predictive of, POCD at 1, 7, and 90 days after the procedure. Our primary hypothesis was that having more episodes of deep sedation (PSI<26) corresponding to burst suppression would be correlated with developing new-onset or worsening POCD.

## Materials and methods

This was a pragmatic, double-blinded observational study of 400 patients aged 65 and older undergoing upper GI endoscopy, lower GI endoscopy, or a combined procedure utilizing propofol sedation at a tertiary-care academic medical center. The study was approved and monitored by the local Institutional Review Board (Protocol No. 17-156).

Patient selection

Subjects were enrolled upon presentation to the Special Tests Unit of the hospital. Table 1 lists the inclusion and exclusion criteria of the study.

**Table 1:** Inclusion and exclusion criteria EGD: Esophagogastroduodenoscopy; ERCP: Endoscopic retrograde cholangiopancreatography; ASA: America Society of Anesthesiologists; EEG: Electroencephalogram; EUS: Endoscopic ultrasound

Inclusion criteria	Exclusion criteria
Patients presenting for endoscopy (EGD, ERCP, EUS, colonoscopy, or combined procedures); patients who are at least 65 years old’ patients who are able to give informed consent; inpatient status: the patient was not expected to be discharged within 24 hours of the procedure.	Patients in whom the anesthesia plan includes the use of midazolam, fentanyl, ketamine, and/or dexmedetomidine as the mainstay of sedation; ASA class 5 patients; emergency cases; patients who, in the opinion of the investigators, would be unable to complete the study for any reason subjects involved in other trials: prisoners known dementia (e.g., Alzheimer’s disease) or reduced mental status for any reason, acute or chronic; known brain tumor or head trauma medications for epilepsy, psychiatric illness, or other agents known to alter the EEG (phenytoin, levetiracetam, amphetamine, aripiprazole, modafinil, haloperidol, selective serotonin reuptake inhibitors, benzodiazepines), if used daily. Sporadic use (e.g., of alprazolam for anxiety attacks) is allowed, as long as it has not been administered on the day of the procedure; substance abuse, i.e., benzodiazepines, opiates, alcohol, and marijuana; severe chronic obstructive pulmonary disease, as evidenced by home O2 use; uncontrolled or severe anxiety requiring daily benzodiazepine administration; history of difficult airways intubated or unconscious patients; patients on methadone or fentanyl patches known unusual or extreme anesthetic requirements; an unusual amount of opioid needed to control pain; morbidly obese patients (BMI > 40) inability to apply the SedLine® brain function monitor strip (scalp or forehead defects); known allergy to clear plastic hospital tape

Management of anesthesia

We instructed the anesthesia providers to provide only propofol for sedation. We instructed them not to administer midazolam, fentanyl, or ketamine in order to standardize the anesthetic unless, in the opinion of the attending anesthesiologist, comfortable or adequate sedation could not be achieved without such adjuvant agents. The propofol infusion was titrated to effect as determined by the anesthesia provider administering the anesthetic. We documented the deepest level of sedation achieved during the procedure using the Richmond Agitation Sedation Scale [10].

Depth of sedation monitoring

We monitored the patients with the SedLine® Brain Function Monitor, software version 2 (Masimo Corporation, Irvine, CA), throughout the case, starting at baseline (before administration of propofol) and stopping at case end. The monitor calculated the parameter known as PSI based on four-channel EEG and artifact rejection algorithms, which consider frequency and phase information from antero-posterior relationships as well as the power and coherence between the hemispheres [11]. The PSI algorithm and monitor have been designed for intraoperative and intensive care monitoring. The monitor was only visible to a research coordinator. We exported the PSI values from the monitor in a digital format, which were available at two-second intervals for the duration of the procedure, except in cases of sensor disconnect, artefactual readings, or other interference. We derived the following PSI parameters for the purposes of exploratory analysis: procedure duration, total duration spent with PSI<26 during the procedure, mean duration spent with PSI<26 during the procedure, number of episodes with PSI<26, and percentage of time spent with PSI<26. Studies have shown that patients undergoing propofol sedation for outpatient colonoscopy frequently reach deep planes of anesthesia, often with burst suppression, corresponding to PSI<26 [12,13]. Burst suppression is associated with coma, general anesthesia, and other pathological states and is a marker for deep cerebral quiescence [14]. 

The patient, gastroenterologist, and anesthesia providers were blinded to the monitor output. The coordinator administering the short blessed test (SBT; see below) was blinded to the details of the anesthetic and the monitor output. Patient demographic data, primary and secondary diagnoses, procedure duration, and hemodynamic and pharmacologic data (i.e., propofol dosing and hemodynamic drugs) were recorded in the EPIC electronic anesthesia record (EPIC Systems, Verona, WI), which we abstracted into a study master data file after the completion of the procedure. 

Cognitive function assessment

We assessed the subjects’ cognitive function via an in-person interview at baseline (pre-procedure) and telephone interviews at 1, 7 (±1), and 90 days after study enrollment. We assessed cognitive function by administering the SBT, which is a validated brief cognitive screening appropriate for in-person and telephone administration [15]. The SBT is also known as the Orientation-Memory-Concentration Test. It provides a summative score ranging between 0 and 28, where 0-4 indicates normal cognition, 5-9 indicates questionable impairment, and ≥10 indicates impairment consistent with dementia. The SBT has a sensitivity of 95% and a specificity of 65% in detecting cognitive impairment in emergency department patients and optimally overlaps with the Mini Mental Status Exam in this population compared with the Ottawa 3DY, Brief Alzheimer’s Screen, and caregiver-completed tests for cognitive dysfunction [15]. A more sophisticated battery of neurocognitive tests was not practical due to the pragmatic nature of our study and the time commitment it would have required on the part of the enrollees. The SBT was administered by a single trained clinical-research coordinator.

Statistical analysis

We summarized baseline characteristics by number (%), mean ± standard deviation (SD), or median (first quartile, third quartile) as appropriate. We used Pearson’s correlation coefficient to assess the association between SBT change at the different time points and the bivariate association between procedure duration and change in SBT at days 1, 7, and 90. We used a linear mixed-effect model for repeated measures, considering random days of change in SBT measures for every individual, to analyze the effect of procedure duration on change in SBT post-baseline under conditions where confounding factors such as gender, age, and other variables were controlled, with the missing outcomes handled under the missing-at-random mechanism. We performed all statistical analyses using SAS 9.4 and R version 4.2.2 for Windows, with conclusions made at the 5% significance level.

## Results

**Figure 1:**
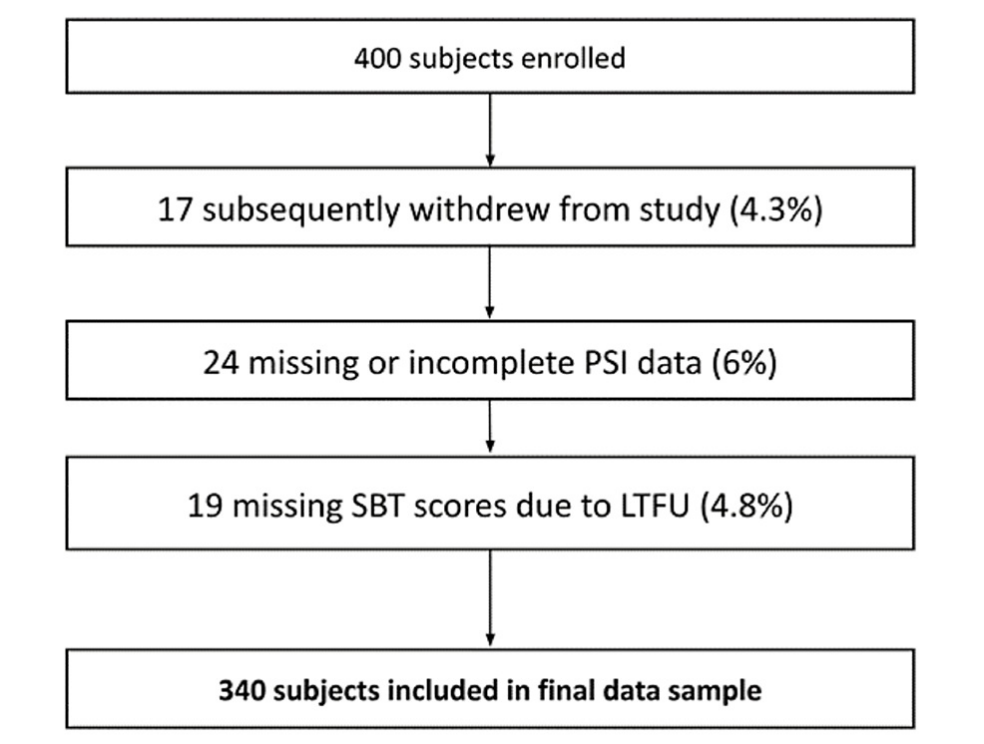
Cohort development flowchart SBT: Short blessed test; LTFU: Lost to follow-up; PSI: Patient state index

After receiving written informed consent, we enrolled 400 subjects in the study. Figure 1 shows the development of the final data sample from the initial cohort, which yielded 340 evaluable subjects.

Table 2 provides the baseline and intraoperative characteristics of the final cohort. All except three subjects were outpatients, and there were no unplanned admissions in the outpatients or readmissions in any of the patients. All patients were sedated with propofol. Eight received 2 milligrams or less of midazolam, and two received supplementary doses of ketamine during their procedure (30 mg and 10 mg). None of the subjects received centrally acting anticholinergics. Two patients received 50 micrograms of fentanyl, and one received 25 micrograms of fentanyl. No other opioids were used.

**Table 2:** Key baseline and intra-procedural characteristics of the study cohort Data are expressed as mean (standard deviation) or count (%) unless otherwise specified. BMI: Body mass index; EGD: Esophagogastroduodenoscopy; EUS: Endoscopic ultrasound; ERCP: Endoscopic retrograde cholangiopancreatography; RASS: Richmond agitation sedation scale; SBT: Short blessed test; mg: Milligrams; mcg: Micrograms.

Characteristics	Value
Baseline	
Male gender	151 (44%)
Mean age (years)	72 (6)
Mean BMI, kg/m^2^	28.93 (5.7)
ASA score:	
I	4 (1%)
II	110 (32%)
III	195 (57%)
IV	31 (9%)
Procedural	
Inpatient	3 (1%)
Type of procedure:	
EGD only	60 (18%)
EGD with EUS	40 (12%)
ERCP	3 (0.9%)
Colonoscopy	156 (46%)
Fecal transplant	1 (0.3%)
Combined EGD/colonoscopy	80 (24%)
Mean procedure duration (minutes)	38.6 (19.5)
Mean propofol dose (mg)	377
Number of patients who received ≤2 mg midazolam:	8 (2.35%)
Number of patients who received ≤30 mg ketamine:	2 (0.59%)
Number of patients who received ≤50 mcg fentanyl:	3 (0.88%)
Deepest level of sedation achieved (lowest RASS score):	
-5	280 (82%)
-4	36 (11%)
-3	8 (2%)
-2	2 (0.6%)
-1	14 (4%)
Mean intraoperative crystalloid (milliliters)	378
Number of patients who received any pressor:	27 (7.9%)
Phenylephrine count/mean dose	23/289 mcg (6.76%)
Ephedrine count/mean dose	8/21.8 mg (2.35%)
Number of patients who received an antihypertensive:	
Hydralazine	1 (0.29%)
Beta blocker	5 (1.47%)

One subject experienced an unplanned intubation during the procedure. There were no other procedural or anesthetic complications. No patients experienced bowel perforation, nausea/vomiting, neurological complications (e.g., stroke, transient ischemic attack, or seizures), deep venous thrombosis, myocardial ischemia or infarction, cardiovascular collapse, death, sepsis, pneumonia, respiratory failure requiring mechanical ventilation, or prolonged awakening (i.e., >30 minutes).

**Figure 2:**
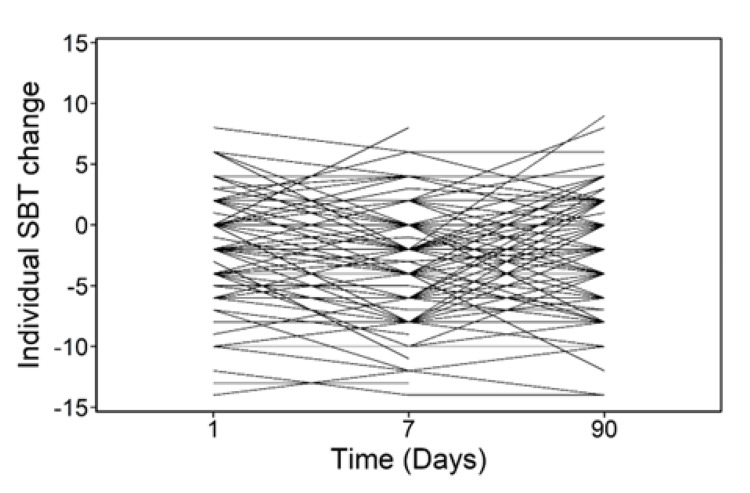
Individual short blessed test (SBT) differences from baseline (day 0). The differences represent subtracting SBT scores at days 1, 7, and 90 from those at day 0 SBT: Short blessed test

**Figure 3:**
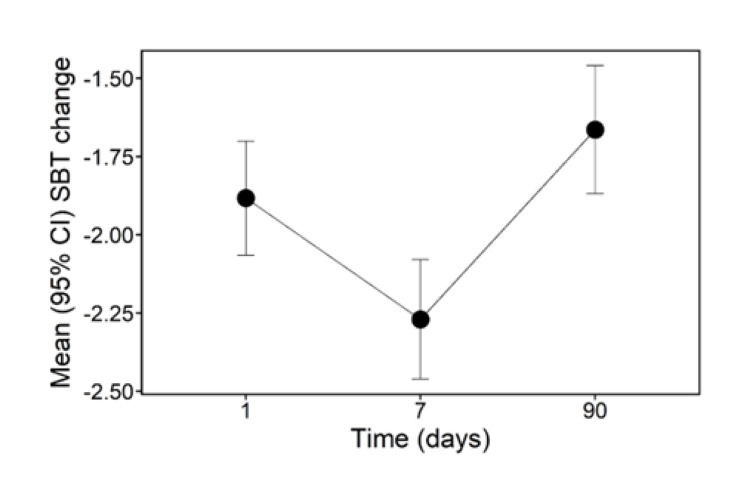
Average short-blessed test (SBT) difference from baseline (day 0). This is the average of the individual differences from baseline at days 1, 7, and 90. The graph illustrates the average trend in SBT change post-baseline for the whole study cohort SBT: Short blessed test, CI: Confidence interval

11% of the subjects had impairment consistent with dementia at baseline, as indicated by a SBT score of ≥10. The corresponding percentage decreased to 2.15% at day 1, 1.3% at day 7, and 2.95% at day 30. The proportion in each SBT category (0-4=normal cognition; 5-9=questionable impairment; and ≥10=impairment consistent with dementia) and the distributions of the mean raw SBT score and the change from baseline are shown in Tables 3, 4. SBT decreased at all time points. Figures 2, 3 show plots of the individual and mean SBT differences from baseline, respectively. Figure 2 indicates that the individual patients started at different values of change in SBT scores, which remained fairly constant over time, whereas Figure 3 indicates that their overall mean SBT score changed significantly at day seven compared to baseline.

**Table 3:** Distribution of the SBT score at baseline, 1, 7, and 90 days SBT: Short blessed test; N: Number of rows with SBT data; SD: Standard deviation

	N	SBT 0-4	SBT 5-9	SBT ≥ 10	Mean	SD
Day 0	340	69.71%	19.12%	11.18%	3.7	3.9
Day 1	325	87.08%	10.77%	2.15%	1.8	2.9
Day 7	307	90.88%	7.82%	1.3%	1.2	2.3
Day 90	271	88.93%	8.12%	2.95%	1.8	2.7

**Table 4:** Distribution of SBT change from baseline at 1 , 7, and 90 days SBT: Short blessed test; N: Number of rows with SBT data; SD: Standard deviation

	N	Mean	SD
Day 1	325	-1.9	3.3
Day 7	307	-2.3	3.3
Day 90	271	-1.7	3.4

Table 3 lists the distribution of SBT scores at baseline and at 1, 7, and 90days after the procedure. Table 4 lists the changes in SBT score from baseline at days 1, 7, and 90.

The correlation between the change in SBT and the pre-defined parameters of PSI (i.e., total duration spent with PSI<26, mean duration spent with PSI<26, number of episodes with PSI<26, and percentage of time spent with PSI<26) was insignificant (all p-values>5%). Table 5 summarizes the results of our linear mixed model analysis. There was a significant drop in SBT score at day seven, indicating improved cognition. Higher age was also significantly associated with the average drop in SBT from baseline. Deep sedation, as evidenced by the number of times when PSI<26, was not predictive of the change in SBT, nor was gender, total propofol dose, or vasoactive drug use during the procedure.

**Table 5:** Linear mixed models PSI: Patient state index, SBP: Systolic blood pressure, SBT: Short blessed test, SE: Standard error, CL: Confidence limits; LCL: Lower confidence limit; UCL: Upper confidence limit

			95 % CL		
Variable	Average change in SBT from baseline	SE	LCL	UCL	p-value
Procedure duration	-0.021	0.012	-0.045	0.002	0.08
Time effect					
Day 7	-0.446	0.133	-0.707	-0.186	0.0008
Day 90	0.137	0.154	-0.165	0.439	0.37
Day 1 (reference)					
Age	-0.067	0.031	-0.128	-0.007	0.0301
Number of times PSI < 26	0.077	0.082	-0.085	0.239	0.35
Ephedrine use	0.721	1.186	-1.607	3.050	0.54
Beta blocker	0.496	1.460	-2.372	3.362	0.73
Propofol dose, mg	0.001	0.001	-0.002	0.003	0.52
Preoperative SBP	-0.002	0.007	-0.017	0.012	0.75
Male	-0.040	0.351	-0.728	0.649	0.91
Hydralazine	1.720	3.474	-5.103	8.543	0.62

## Discussion

Principal findings

Our main findings were that patients undergoing propofol sedation for GI endoscopies experienced a decline in their SBT scores at 1-, 7-, and 90-days post-procedure, which was statistically significant at day seven (p<0.05). Depth of sedation, as documented by the PSI index, was not significantly associated with a decline in SBT score. Age was significantly associated with a decline in SBT score. For every year of age increase, the average change in SBT score was −0.067. The procedure duration in minutes trended towards an association with a lower average SBT score but did not reach statistical significance. The baseline SBT score was not associated with changes in SBT score at days 1, 7, and 90. The incidence of cognitive impairment consistent with dementia was 11.18% at baseline, 2.15% at 1 day, 1.3% at day 7, and 2.95% at day 90.

The incidence of POCD in our patient population at 90 days was lower than that reported in patients undergoing sedation for coronary angiography but not zero, as reported for controls not undergoing any sedation [4]. The lack of a significant association between SBT outcome and our pre-defined parameters of PSI ran contrary to our hypothesis.

Mechanisms underlying observations

The low incidence of POCD in this study-1.2% at 7 days-may be explained by the lack of physiological stress associated with gastrointestinal endoscopies and their underlying physiology. In one study, POCD in a cohort of patients undergoing coronary angiography, which is a similarly low-stress procedure, was 21% [4]. This much higher incidence of POCD for coronary angiography patients may be explained by the stress of myocardial ischemia and the associated impairment in cerebral blood flow or co-existent cerebrovascular disease.

Burst suppression (PSI<26) was not associated with a decline in cognitive function, as measured by the SBT score, which may have been due to the short length of endoscopic procedures. In addition, endoscopic procedures do not cause surgical stimulation (i.e., there is no skin incision) and may allow for a smoother titration of anesthetic drugs, which may lead to fewer or shorter periods of burst suppression.

In many studies where processed EEG is used to guide anesthetic delivery, the procedures are longer and are associated with higher levels of surgical stimulation, and the patient is exposed to higher doses of anesthetics and/or opiates. In one cohort study of patients undergoing non-cardiac surgery, patients spent a median time of 50-90 min in burst suppression [16]. 

The fact that the SBT score decreased at all time points after baseline could have been the effect of practice/increased familiarity with the SBT. This may also suggest that the baseline tests were undertaken in patients who were anxious, rushed, or distracted due to the impending procedure and did not provide a true indication of cognitive status. Further research may consider baseline testing before the day of the procedure. Finally, unknown variables that were not in our linear mixed model could have been associated with a lower SBT score.

A recent umbrella review of observational data assessing perioperative risk factors for POCD reports that as many as 31 factors are associated weakly with the development of POCD [17]. In this review, however, no risk factor or set of risk factors was consistently associated with POCD, including comorbidities such as diabetes, hypertension, and heart failure. While patient comorbidities may certainly play a role in the development of POCD, it is unclear what effect these comorbidities would have on the current study.

Previous studies on the predictive value of processed EEG parameters for POCD

A Cochrane database review from 2018 aimed to assess whether the use of processed EEG or auditory evoked potential as guides to anesthetic delivery can reduce the risk of POCD in non-cardiac surgical or non-neurosurgical adult patients undergoing general anesthesia compared with the standard practice where only clinical signs are used [18]. The authors reported on six randomized control trials and found that there was moderate-quality evidence that optimized anesthesia guided by processed EEG indices could reduce the risk of POCD in patients aged 60 years or older.

Deiner and colleagues conducted a single-center cohort trial examining the use of several processed EEG parameters (Bispectral Index, raw, and processed EEG) to predict POCD 30 days after surgery. The incidence of POCD in this cohort was 27%, much higher than that (1.3%) at seven days in the present study [19].

Most studies examining the use of processed EEG to reduce the risk of POCD are focused on general anesthesia with volatile anesthetics. The present study examined the relationship between propofol sedation and POCD in patients undergoing GI endoscopies. Using processed EEG to optimize propofol sedation may lessen the amount of time in deep planes of anesthesia or burst suppression [13,20]. One recent single-center study using PSI generated by the Masimo SedLine® Brain Root Function monitor found that a significant number of patients had processed EEG patterns consistent with general anesthesia or burst suppression despite a plan for moderate to deep sedation [12]. However, in this study, the total dose of propofol did not differ between groups and had no effect on the SBT result.

Clinical implications

The risk of POCD in elective GI endoscopies performed with propofol sedation is low. Our study noted a robust recovery of cognitive function after propofol anesthetics for endoscopy procedures. Future studies should consider whether extending propofol anesthesia to procedures performed with other types of anesthesia could be associated with a lower incidence of POCD.

Processed EEG may still be used in a subset of geriatric patients who are at risk for adverse outcomes as a result of hypotension due to propofol administration. These risks include patients who are critically ill and those with myocardial ischemia, stroke, and/or active bleeding.

Unfortunately, there are no established strategies for decreasing the risk of POCD in the elderly population. This is why the identification of risk factors is an important research endeavor. Pharmacologic interventions, for example, the use of dexmedetomidine, may have an effect on the rate of POCD, but this relationship is not well established, and the mechanism of its effect on POCD is unclear.

Methodological considerations and potential weaknesses of the study

Concerns with the measurement of POCD have been described in detail and include a limited generalizability due to the under-representation of lower-education individuals, minority groups, and languages other than English [21]. Another consideration is whether a change in cognitive function represents an expected change or a change attributed to the procedure. For this specific study, one would not expect a significant change in cognition during the short duration of the procedure.

The limitations of the study include instances of deviation from protocol. Thirteen patients received medications that may have altered their sedation: eight received midazolam, two received ketamine, and three received fentanyl. No patients in our study sample were taking buprenorphine or naloxone for opioid use disorder, as these medications are less common in the geriatric population. Additionally, although the SBT is pragmatic and easy to administer, it is not considered a standard tool for assessing postoperative cognitive function. A battery of tests measuring semantic/language fluency, visuoperception, and motor function, as well as changes in memory and executive measures that are known to be abnormal in POCD, may have been more sensitive and specific for detecting POCD, but this would have been impractical in our setting [22,23]. Another potential weakness of this study was that a neurophysiologist was not available to review the raw EEG tracings for episodes of burst suppression corresponding to our selected value of PSI<26. We did not have a comparator group consisting of age- and risk-matched controls who did not undergo a procedure. Since the inception of this study, digital solutions for cognitive research, such as the Digital Clock Drawing Test, have been introduced and validated against established tools such as the Mini Mental Status Exam [24-27]. In the future, such tools may reduce the time and resources required to carry out research on postoperative cognitive function.

## Conclusions

In our cohort, the observed incidence of cognitive dysfunction after gastroenterology procedures with propofol sedation was much lower than has been documented for other similar procedures. These results suggest that, for most patients, undergoing these procedures with propofol sedation does not lead to cognitive dysfunction. Older patients had a greater decline in cognitive function after the procedure, and future research should aim to better characterize the risk of cognitive dysfunction in older patients. The level of sedation, as documented by PSI, was not associated with cognitive dysfunction after the procedure, as measured by the SBT.
